# The Versatile Applications of Triple‐Wavelength Diode Laser (810, 940, and 1060 nm) in Aesthetic Treatments, Follicular Disorders, and Chronic Inflammatory Conditions in the Asian Population: Case Report Collection

**DOI:** 10.1111/jocd.70231

**Published:** 2025-06-04

**Authors:** Anuj Pall

**Affiliations:** ^1^ Escallent Institute of Lasers and Aesthetic Medicine (EILAM) Gurugram India

**Keywords:** blend wavelength, dark skin types, diode laser, hair removal, hidradenitis suppurativa, trichostasis spinulosa, pilonidal sinus

## Abstract

**Background:**

Single‐wavelength lasers (755 or 810 nm) are widely used to remove unwanted hair. Recently, combined‐wavelength diode lasers have been introduced to improve the safety of darker skin types, owing to their varying absorption spectra and penetration depths. However, their application beyond hair removal remains unclear.

**Aims:**

This study aimed to evaluate the efficacy of a high‐power triple‐wavelength diode laser (810, 940, and 1060 nm) in treating hirsutism, inflammatory follicular disorders, and aesthetic concerns such as hairline enhancement in darker skin types.

**Patients/Methods:**

This retrospective study was conducted at the Escallent Institute of Lasers & Aesthetic Medicine (EILAM), Gurugam, India, using a Primelase device with a triple‐wavelength diode laser. The study involved adults with skin type IV who presented with facial hirsutism and follicular disorders, including trichostasis spinulosa, pilonidal sinus disease, and hidradenitis suppurativa. Efficacy was assessed using hair counting and the Global Aesthetic Improvement Scale (GAIS), whereas safety was evaluated based on reported adverse effects, such as pain, erythema, and edema.

**Results:**

The GAIS scores indicated a mean improvement of 3.4 ± 0.4 out of 4, representing a 50%–75% improvement. Hair counting revealed a statistically significant hair reduction of 82.9% ± 15.4%, with reductions ranging from 56% to 100%. No adverse events were observed.

**Conclusion:**

The high‐power triple‐wavelength diode laser demonstrated both efficacy and safety in treating facial hirsutism and other follicular disorders as well as aesthetic concerns such as hairline enhancement, particularly in darker skin tones.

## 1 Introduction

Among various light‐based technologies, high‐power diode lasers have become a well‐established technique in cosmetic dermatology, with applications in hair removal [1], acne treatment [2, 3] and the treatment of pigmented [4, 5] and vascular lesions [6]. However, their use for other aesthetic purposes, such as hairline enhancement, facial hirsutism, or the treatment of hair follicle‐related disorders, such as trichostasis spinulosa (TS), as well as chronic inflammatory conditions, such as pilonidal sinus disease (PSD) and hidradenitis suppurativa (HS), is relatively underreported, particularly in populations with darker skin types (Fitzpatrick IV–VI). These treatments rely on the same principle as hair removal for hirsutism [7] involving selective thermal heating of the hair follicle through melanin absorption.

The most recognized laser technologies for hair removal include single‐wavelength lasers, such as alexandrite (755 nm), Nd:YAG (1064 nm), and high‐power diode lasers at 810 nm. In dark‐skinned individuals, alexandrite and diode lasers may pose challenges because of their high melanin absorption, which increases the risk of adverse effects. Nd:YAG lasers, with their deeper penetration and reduced melanin absorption, are typically favored for darker skin, although their efficacy may be lower than that of other lasers [8, 9].

To enhance efficacy while minimizing adverse events in darker skin types, high‐power diode lasers combining wavelengths of 755, 810, and 1064 nm have been introduced [10, 11]. These multiwavelength lasers target melanin and penetrate deeper into the skin, thereby optimizing absorption in darker skin types. However, shorter wavelengths (e.g., 755 and 810 nm) still raise safety concerns owing to their high melanin absorption. Alternative approaches using longer wavelengths, such as 810, 940, and 1060 nm, have emerged [12]. This approach aims to improve the penetration depth and enhance absorption by other chromophores such as water and hemoglobin. Longer wavelengths allow for the application of higher fluences in darker skin, thereby increasing efficacy without compromising safety [13, 14].

Building on the positive outcomes observed in general hair removal using a triple‐wavelength diode laser, this report evaluates the efficacy and safety of a high‐power diode laser device emitting wavelengths of 810, 940, and 1060 nm in additional aesthetic and dermatological applications. These conditions include hairline enhancement, facial hirsutism, trichostatic spinulosa (TS), pilonidal sinus disease (PSD), and hidradenitis suppurativa (HS), particularly in Indian patients.

## 2 Materials and Methods

### 2.1 Population and Device

Nine patients were treated at the Escallent Institute of Lasers & Aesthetic Medicine (EILAM), Gurugam, India, between 2018 and 2022. The study included five women and four men aged between 19 and 31 years, all with Fitzpatrick skin phototype IV. Four of the nine participants were diagnosed with pilonidal sinus disease, hidradenitis suppurativa, trichostasis spinulosa, and hirsutism, respectively, whereas five of the other patients received treatment for aesthetic concerns such as forehead, beard, and eyebrow reshaping.

The treatments were performed using a Primelase device (Sinclair High Technology Products SLU, Barcelona, Spain), which utilizes a 4000 W triple‐wavelength diode laser (810, 940, and 1060 nm) with a 20 × 9 mm (2.7 cm^*2*^) spot applicator. Detailed information on patient demographics and treatment specifics is presented in Table 1.

**TABLE 1:** Population characteristics and treatment configurations.

#	Gender (age)	Treated indication	Area (hair characteristics)	Fluence range (J/cm^2^)	Sessions (interval [weeks])
1	Male (27)	HE	Forehead (dark, coarse)	17–20	6 (8–12)
2	Female (26)	HE	Forehead (dark, coarse and fine)	17–19	5 (8–12)
3	Female (20)	HE	Eyebrow (dark, coarse/find)	18–20	6 (6–8)
4	Female (28)	HE	Forehead (dark, coarse)	18	4 (8–10)
5	Male (20)	HE	Beard (dark, coarse/fine)	19–22	6 (8–12)
6	Female (23)	FH	Face (dark, coarse)	18–22	10 (6–8)
7	Male (31)	PSD/HR	Buttocks (dark, coarse)	17–18	5 (8–12)
8	Male (19)	TS	Nose (light, fine)	20–22	5 (8–12)
9	Female (26)	HS	Armpit (dark, coarse/fine)	18–20	8 (8–12)

*Abbreviations:* FH, facial hirsutism; HE, hairline enhancement; HR, hair removal; HS, Hidradenitis suppurativa; PSD, pilonidal sinus disease; TS, Trichostasis spinulosa.

### 2.2 Inclusion and Exclusion Criteria

The inclusion criteria were adult males and females with Fitzpatrick skin types IV to V, unwanted facial hair, or hair follicle‐related dermatological conditions, who had not undergone prior treatment in the affected areas. Participants were required to agree to adhere to all study protocols and recommendations. The exclusion criteria included pregnancy, lactation, age under 18 years, use of photosensitive medication, photosensitive disorders, active skin diseases, and the presence of lesions or tattoos in the treatment area.

### 2.3 Treatment Procedure

Before each session, the treatment area was cleaned and shaved, followed by the application of a cooling gel. Participants wore protective eye shields throughout the procedure. The triple‐wavelength diode laser was applied using single pulses in stamping mode with a frequency of 1 Hz and a pulse duration of 30 ms. The cold sapphire tip ensured constant epidermal cooling by maintaining contact with the skin. No topical anesthesia was administered. The total number of treatment sessions ranged from 4 to 10, with intervals of 6–12 weeks, depending on the treatment conditions (Table 1). All treatments were performed by a dermatologist using recommended parameters for the respective skin and hair types.

For patients with active inflammatory conditions such as HS and PSD, inflammation was controlled with medical management prior to laser treatment. In patients with HS, adalimumab (Humira) was administered subcutaneously, and once the inflammation subsided, laser treatment was commenced. Patient 6, in addition to PSD treatment, underwent general hair removal from the buttocks. Patient 5, who was hirsutic, was prescribed aldactone and oral contraceptives by an endocrinologist to address the underlying hormonal condition.

### 2.4 Efficacy and Safety Evaluation

Safety assessments were conducted by monitoring erythema, edema, and other adverse effects during and immediately after each session. The efficacy of the treatment was evaluated by analyzing paired pre‐ and posttreatment digital photographs, which were assessed by two independent medical researchers. Qualitative results were determined using the Global Aesthetic Improvement Scale (GAIS), with a score of 0 indicating no results (a reduction of 0%–6%), 1 representing poor improvement (a reduction of 0%–25%), 2 representing average improvement (a reduction of 25%–50%), 3 representing good improvement (a reduction of 50%–75%), and 4 representing excellent improvement (a reduction of 75%–100%). Quantitative results were obtained by counting the number of hair follicles in a 4 × 3 cm section of the treatment area.

All participants were informed about the treatment procedure and written informed consent was obtained. Data were collected anonymously and patient confidentiality was maintained throughout the study.

### 2.5 Statistical Analysis

Mean values and standard deviations were calculated for the quantitative variables. Results are presented as mean value ± standard deviation (SD). The statistical significance of the before and after results was determined with a Student's two‐tailed paired *t*‐test, and statistical analysis was performed using Microsoft Excel 365.

## 3 Results

**FIGURE 1:**
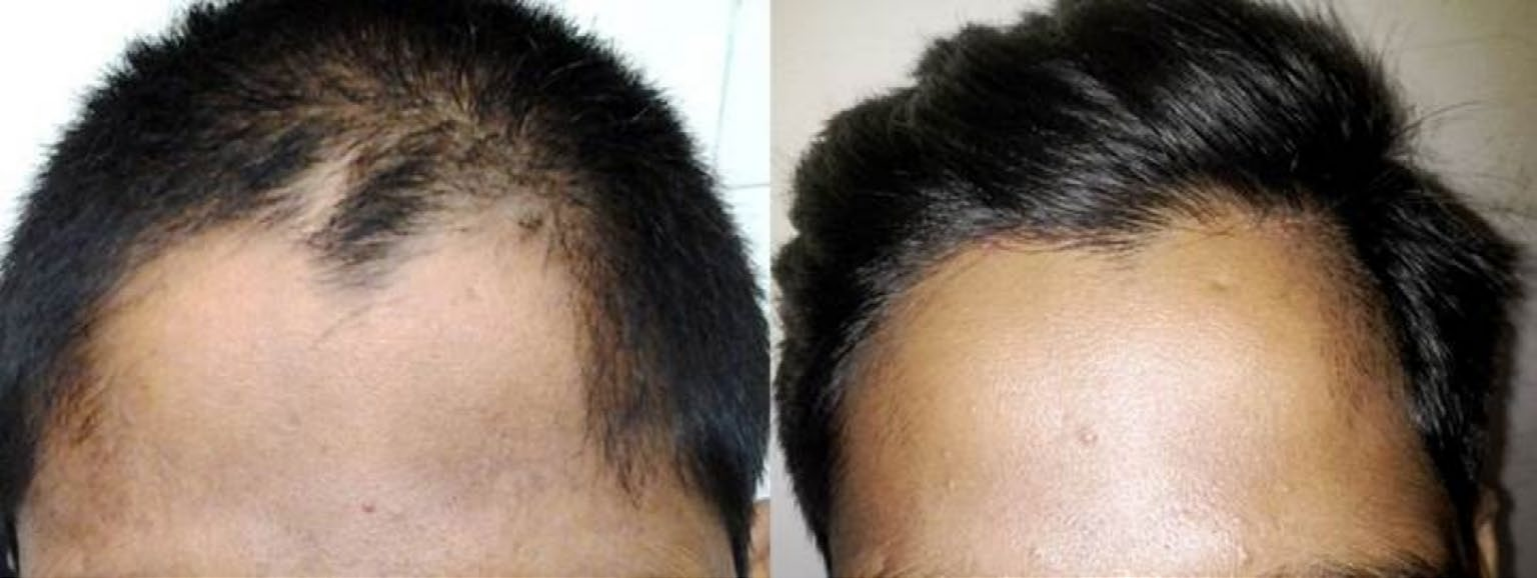
Male, 27. Hairline enhancement treatment of the forehead. Left: before treatment; right: after 6 sessions every 8–12 weeks with a fluence range of 17–20 J/cm^2^ in each session.

**FIGURE 2:**
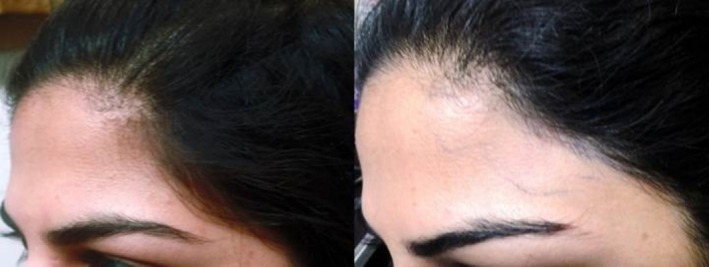
Female, 26. Hairline enhancement treatment of the forehead. Left: before treatment; right: after 5 sessions every 8–12 weeks with a fluence range of 17–19 J/cm^2^ in each session.

**FIGURE 3:**
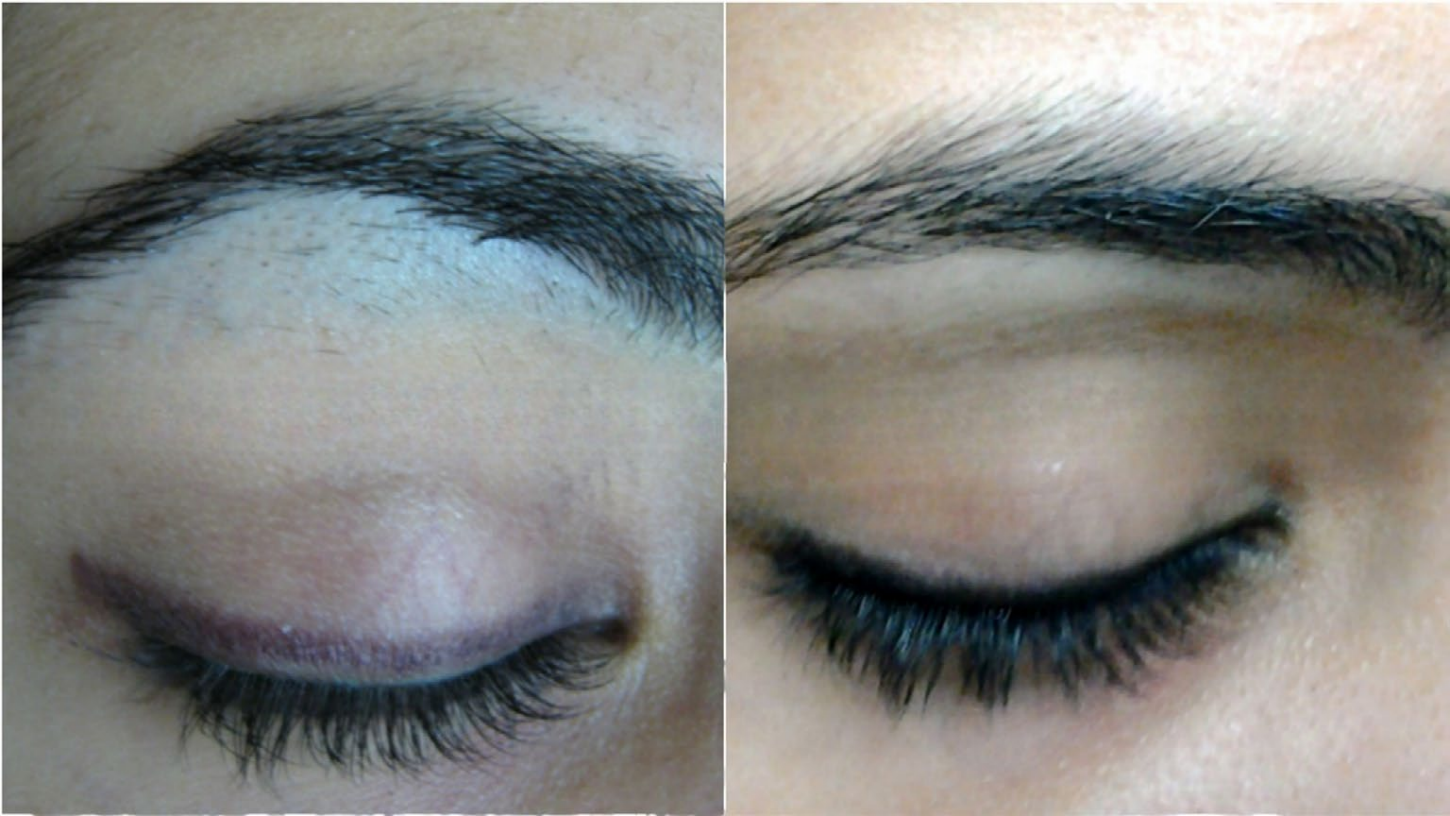
Female, 20. Hairline enhancement treatment on the eyebrow. Left: before treatment; right: after 6 sessions every 6–8 weeks with a fluence range of 18–20 J/cm^2^ in each session.

**FIGURE 4:**
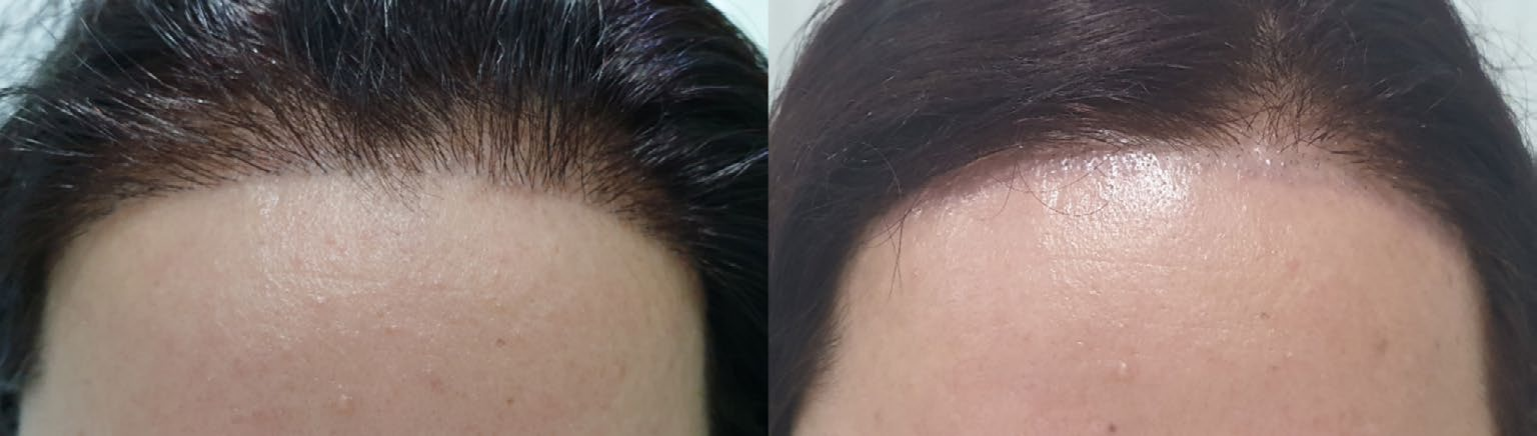
Female, 28. Hairline enhancement treatment on the forehead. Left: before treatment; right: after 4 sessions every 8–10 weeks with a fluence range of 18 J/cm^2^ in each session.

**FIGURE 5:**
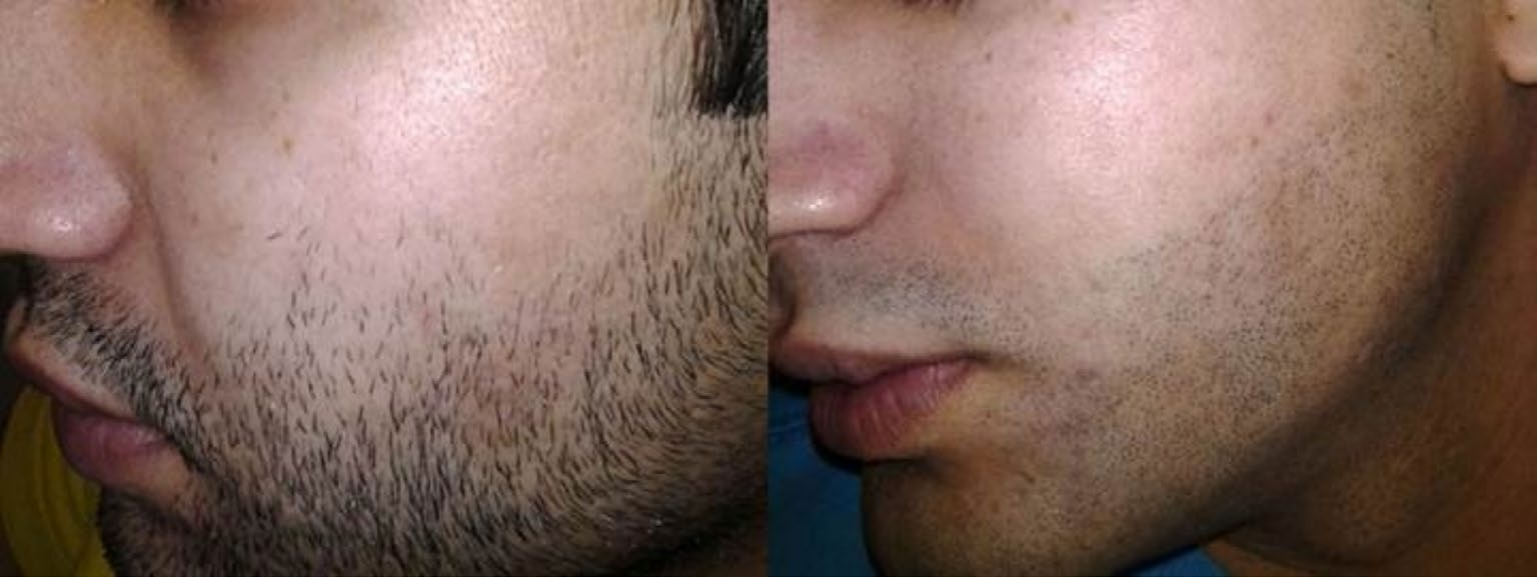
Male, 20. Hairline enhancement treatment of the beard. Left: before treatment; right: after 6 sessions every 8–12 weeks with a fluence range of 19–22 J/cm^2^ in each session.

**FIGURE 6:**
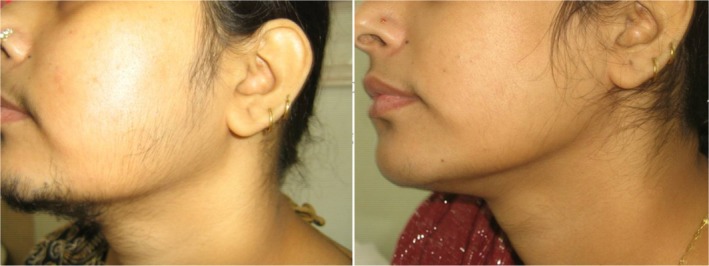
Female, 23. Hirsutism treatment of the face. Left: before treatment; right: after 10 sessions every 6–8 weeks with a fluence range of 18–22 J/cm^2^ in each session.

**FIGURE 7:**
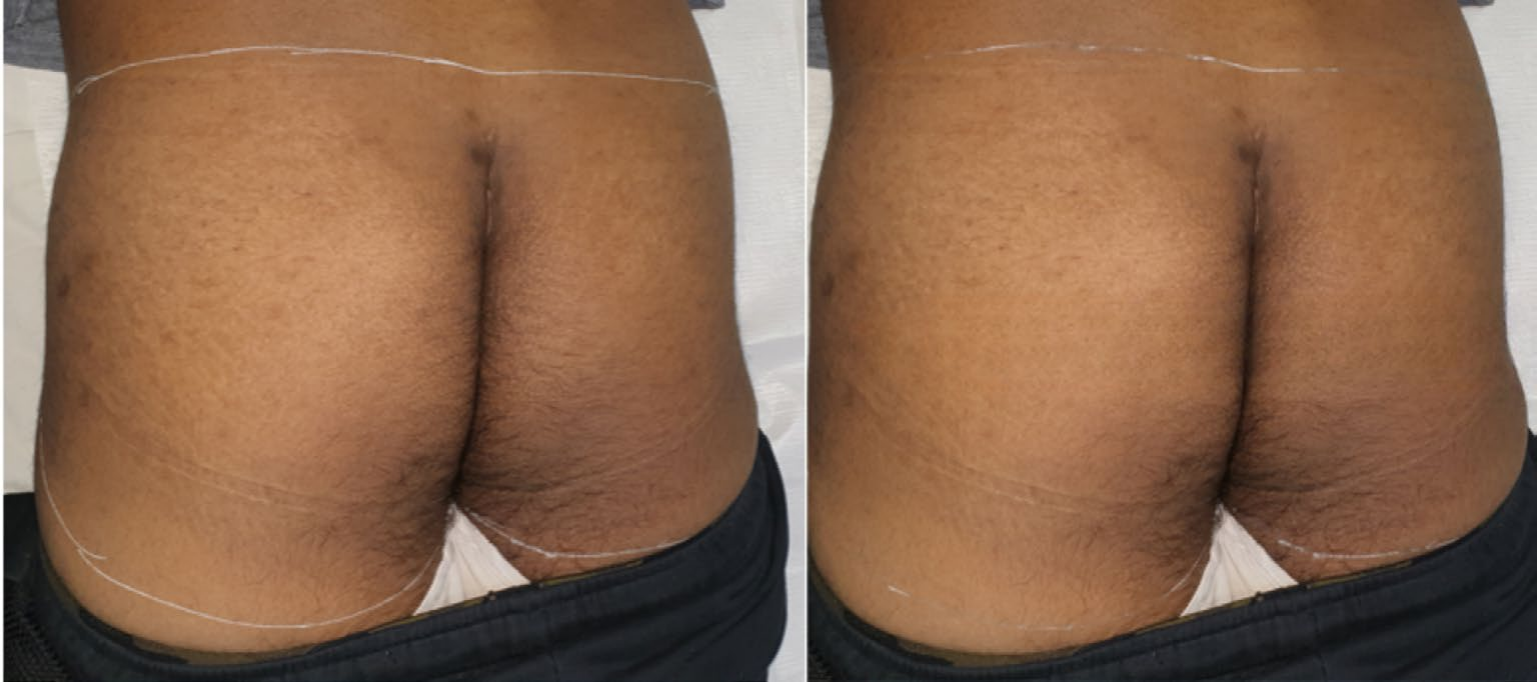
Male, 31. Pilonidal sinus disease and hair removal treatments on buttocks. Left: before treatment; right: after 5 sessions every 8–12 weeks with a fluence range of 17–18 J/cm^2^ in each session.

**FIGURE 8:**
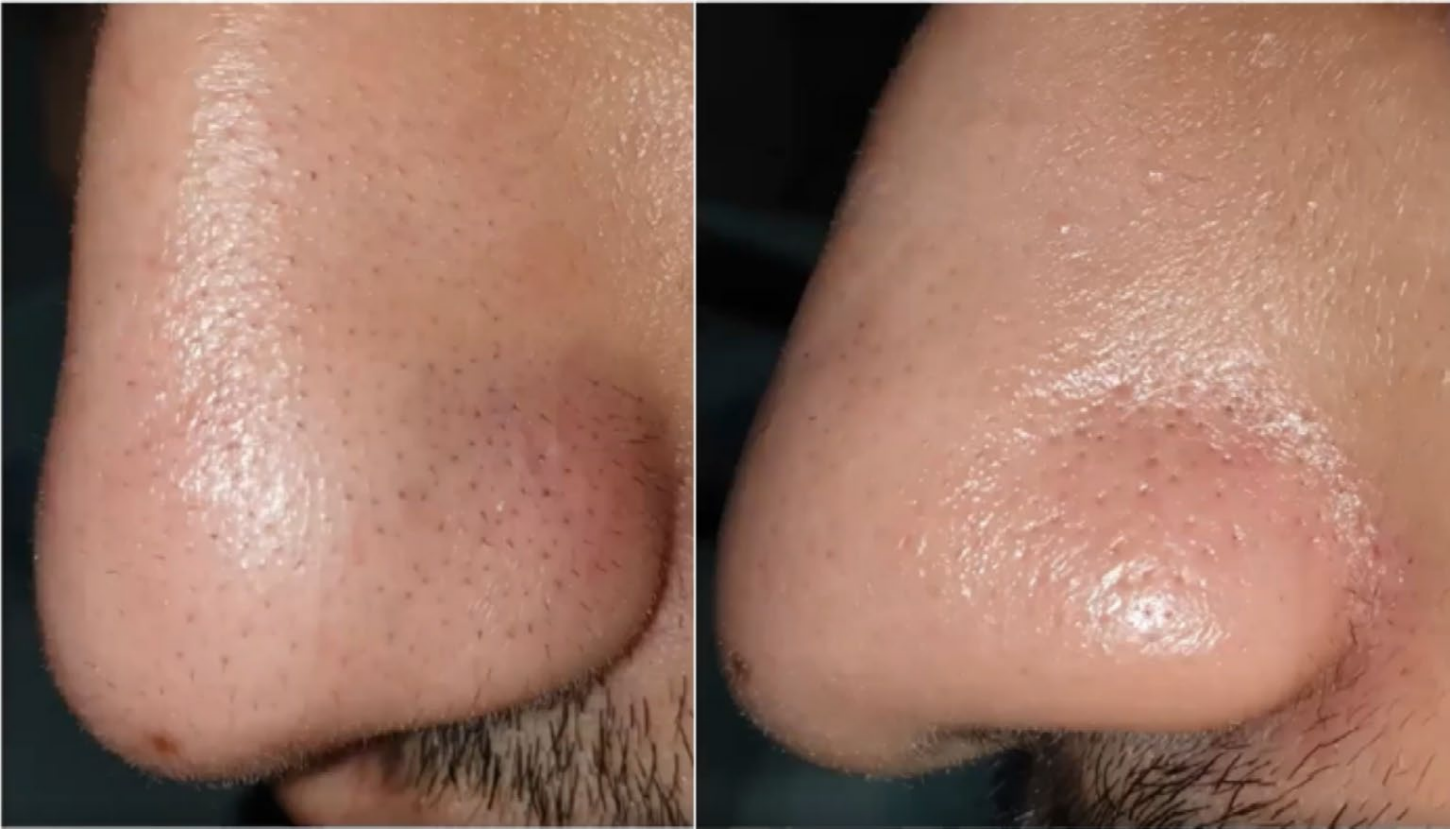
Male, 19. Trichostasis spinulosa treatment on the nose. Left: before treatment; right: after 5 sessions every 8–12 weeks with a fluence range of 20–22 J/cm^2^ in each session.

**FIGURE 9:**
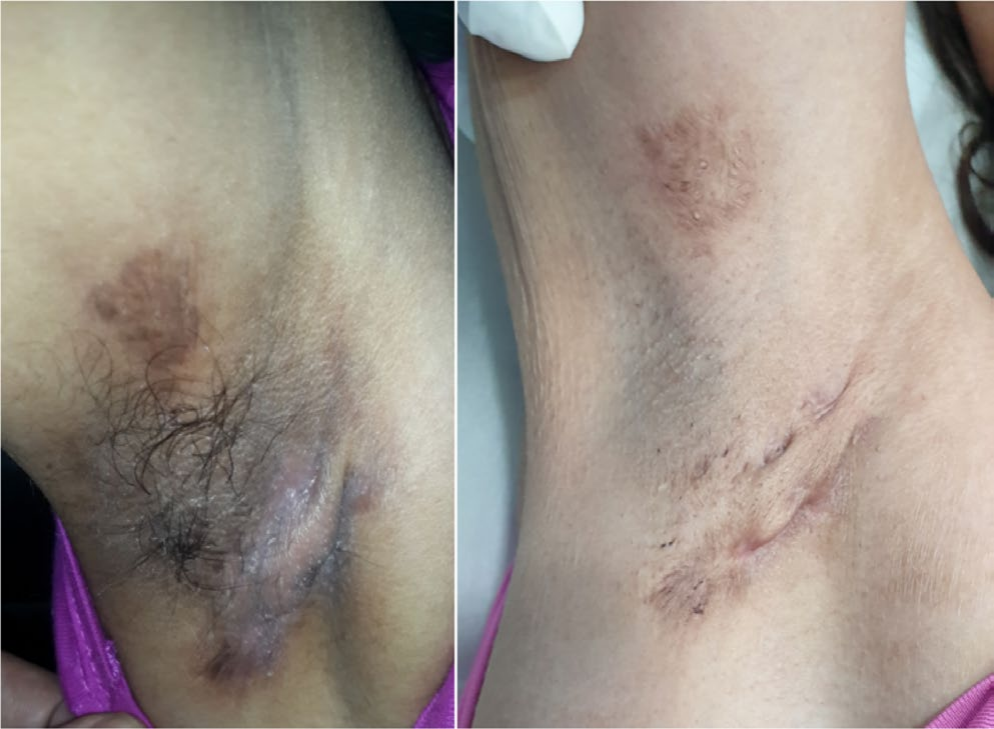
Female, 26. Hidradenitis suppurativa treatment of the armpit. Left: before treatment; right: after 8 sessions every 8–12 weeks with a fluence range of 18–20 J/cm^2^ in each session.

**FIGURE 10:**
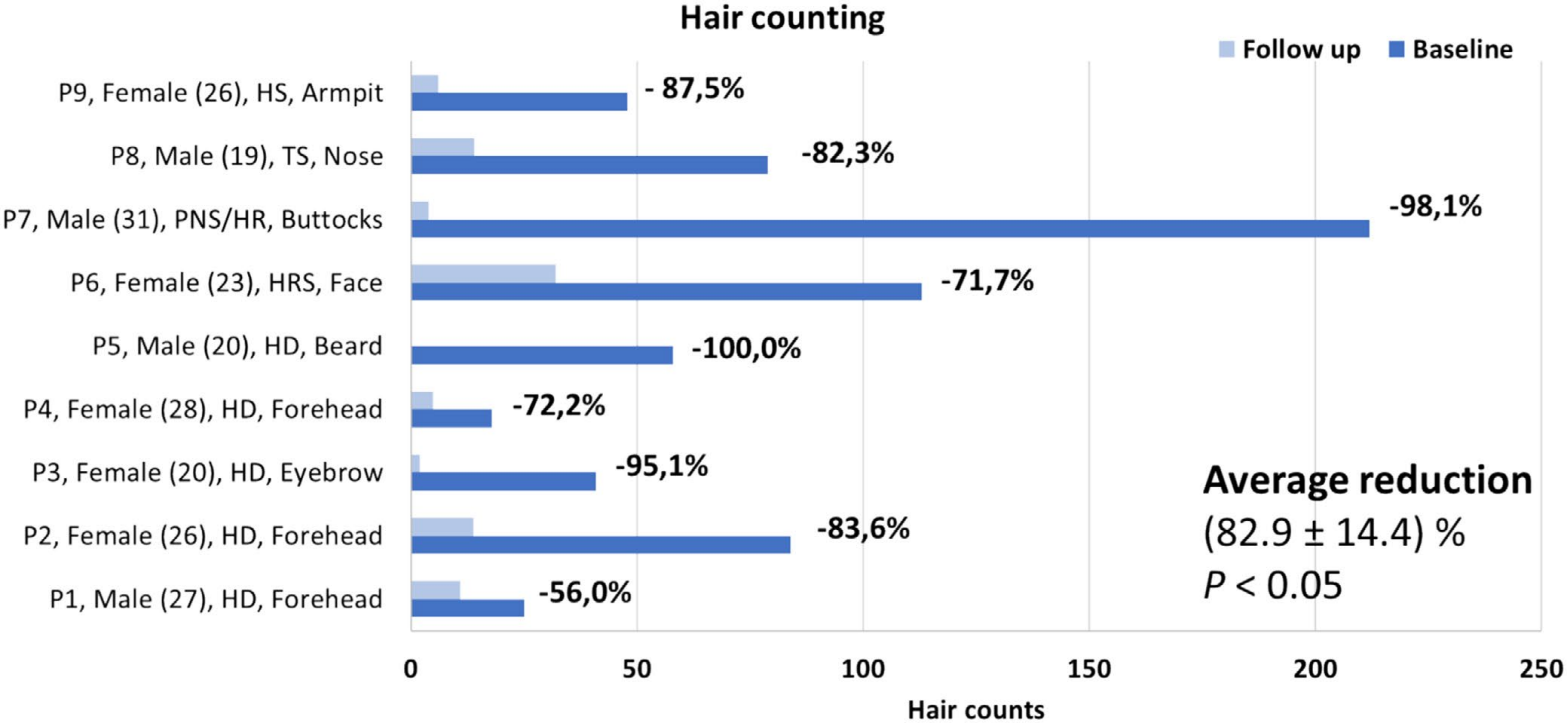
Individual hair counting results of each subject at baseline and follow‐up.

Figures 1, 2, 3, 4, 5, 6, 7, 8, 9 show before and after photographs of each patient. The GAIS value of the researchers resulted in a mean value of 3.5 ± 0.4, which implies an average improvement between 50% and 75%. Hair counting resulted in a reduction of (82.9 ± 15.4)% with a minimum reduction of 56.0% and a maximum of 100.0%. The results were considered statistically significant (*p* < 0.05). Figure 10 shows the hair count results for each participant. Mild and moderate erythema and edema were observed after each treatment, and recovery was almost immediate or occurred 48 h after treatment. No other adverse events were observed.

## 4 Discussion

This study aimed to evaluate the efficacy and safety of a high‐power triple‐wavelength diode laser (810, 940, and 1060 nm) for aesthetic hairline enhancement and dermatological hair removal‐related applications, including hirsutism, PSD, TS, and HS, specifically in the Indian population.

For individuals with darker skin types (IV–VI), standard laser hair removal treatments using alexandrite (755 nm) and diode (810 nm) lasers have clinical limitations because of the high absorption of laser energy by skin melanin, which can lead to potential side effects or reduced effectiveness, as laser energy must be reduced to prevent skin damage [8, 15]. In contrast, diode lasers employing a combination of wavelengths have emerged as a promising option for darker skin types. However, traditional combinations, such as 755, 810, and 1064 nm, may still be suboptimal because shorter wavelengths (755 and 810 nm) remain highly susceptible to melanin absorption and offer limited energy penetration [10, 16].

The combination of longer wavelengths (810, 940, and 1060 nm) used in this study targeted melanin as well as other chromophores such as water and hemoglobin, enhancing skin penetration and facilitating more effective heating and damage to the hair follicle. Previous studies demonstrated the efficacy and safety of this wavelength combination for hair removal from individuals with darker skin [12, 13]. In the present study, significant hair reduction was observed in Indian subjects with Fitzpatrick skin type IV, with a mean reduction of 82.9% ± 15.4%, and no occurrence of serious or unexpected adverse effects, aligning with findings from prior research [14]. Additionally, the GAIS results indicated notable improvements, ranging between 50% and 75%, further affirming the treatment's efficacy and safety.

Participants 1–6 (Figures 1, 2, 3, 4, 5, 6) underwent aesthetic hairline enhancement and facial hirsutism treatments, achieving a significant reduction in hair (79.0% ± 18.2%) and an average GAIS score of 3.5 ± 0.4 out of 4. It is important to note that hairline enhancement in patients with dark, coarse hair and dark skin can pose safety concerns owing to the high melanin content in both the skin and hair. Nevertheless, the favorable outcomes observed indicate that selective heating provided by the triple‐wavelength diode laser effectively reduces hair without adverse effects, particularly in areas with frequent and challenging hair presence and reappearance. Hairline enhancement has been demonstrated to be effective in patients with recent hair transplants, where the hairline has been naturalized (Figure 2).

The results for other, less common indications are also noteworthy. PSD typically arises from excessive hair growth in the sacrococcygeal region. Laser treatment, either postoperatively or as a standalone therapy, is the primary modality for managing PSD. Numerous clinical studies have reported low morbidity and reasonable recurrence rates with diode laser treatments [17, 18] as well as with other laser types [19, 20]. Subject 7 (Figure 7) was treated with the combined‐wavelength diode laser and showed successful results, including an almost complete hair reduction in the buttock area, which is critical for PSD management.

Regarding TS, a common yet often underdiagnosed follicular disorder characterized by the retention of successive telogen hairs in the hair follicle, subject 8 (Figure 8) exhibited significant improvement with an 82.3% hair reduction and received the highest GAIS score assigned by the researchers. These findings are consistent with those of other studies conducted in dark‐skinned Asian patients using single‐wavelength diode lasers, underscoring the efficacy of triple‐wavelength diode lasers for treating TS [21, 22].

Hidradenitis suppurativa (HS) is a chronic inflammatory disease of the pilosebaceous follicles that typically presents as nodules, abscesses, or suppurative fistulas in the body folds, primarily in the armpits. The recent increase in laser utilization for HS can be attributed to its favorable outcomes and fewer complications compared with conventional surgical and systemic treatments, which often involve greater difficulty and longer recovery times [23]. Subject 9 (Figure 9) showed a significant improvement, achieving the highest GAIS score, thereby demonstrating the efficacy of the triple‐wavelength diode laser in treating HS. Moreover, no adverse effects were observed, and the results were comparable to those obtained using other laser technologies.

In conclusion, the high‐power triple‐wavelength diode laser (810, 940, and 1060 nm) demonstrated high efficacy in hair reduction and a strong safety profile in treating various aesthetic and dermatological conditions in individuals with darker skin types. Its versatility extends beyond hair removal and effectively addresses conditions such as hirsutism, PSD, TS, and HS, thereby offering dark‐skinned patients a reliable and multifaceted treatment option.

However, the primary limitation of this study is its small sample size. To further validate these findings, larger comparative studies with more participants, diverse skin types, various anatomical treatment areas, and extended follow‐up periods are necessary. Such studies would provide a more comprehensive understanding of the effects and potential benefits of this unique combination of lasers.

## 5 Conclusion

This study demonstrated that a 4000 W triple‐wavelength high‐power diode laser (810, 940, and 1060 nm) is highly effective and safe for facial hirsutism and the removal of unwanted hair. Additionally, it presents a promising option for treating hair follicle‐related disorders such as trichostasis spinulosa, pilonidal sinus disease, and hidradenitis suppurativa in individuals with darker skin types. The versatility and safety of this laser system make it a valuable tool in cosmetic dermatology, particularly for populations with darker skin tones where traditional laser treatments may pose higher risks.

## Ethics Statement

All the study procedures were conducted in accordance with the Declaration of Helsinki. The author confirms that ethical review committee approval is not necessary as the study device is already a CE‐marked device.

## Conflicts of Interest

The author declares no conflicts of interest.

## Data Availability

The data supporting the findings of this study are available from the corresponding author upon reasonable request.
